# Spatial abundance and clustering of *Culicoides* (Diptera: Ceratopogonidae) on a local scale

**DOI:** 10.1186/1756-3305-6-43

**Published:** 2013-02-22

**Authors:** Carsten Kirkeby, René Bødker, Anders Stockmarr, Peter Lind

**Affiliations:** 1 National Veterinary Institute, Technical University of Denmark, Bülowsvej 27, Frederiksberg C, DK-1870, Denmark; 2 Institute of Informatics and Mathematical Modelling, Technical University of Denmark, Asmussens Allé, DTU, Building 305, Lyngby, DK-2800, Denmark

**Keywords:** *Culicoides*, Spatial clustering, Local scale abundance, Abundance modeling, Spatial autocorrelation, Bluetongue, Schmallenberg virus

## Abstract

**Background:**

Biting midges, Culicoides, of the Obsoletus group and the Pulicaris group have been involved in recent outbreaks of bluetongue virus and the former was also involved in the Schmallenberg virus outbreak in northern Europe.

**Methods:**

For the first time, here we investigate the local abundance pattern of these two species groups in the field by intensive sampling with a grid of light traps on 16 catch nights. Neighboring trap catches can be spatially dependent on each other, hence we developed a conditional autoregressive (CAR) model framework to test a number of spatial and non-spatial covariates expected to affect Culicoides abundance.

**Results:**

The distance to sheep penned in the corner of the study field significantly increased the abundance level up to 200 meters away from the sheep. Spatial clustering was found to be significant but could not be explained by any known factors, and cluster locations shifted between catch nights. No significant temporal autocorrelation was detected. CAR models for both species groups identified a significant positive impact of humidity and significant negative impacts of precipitation and wind turbulence. Temperature was also found to be significant with a peak at just below 16 degrees Celcius. Surprisingly, there was a significant positive impact of wind speed. The CAR model for the Pulicaris group also identified a significant attraction to the smaller groups of sheep placed in the field. Furthermore, a large number of spatial covariates which were incorrectly found to be significant in ordinary regression models were not significant in the CAR models. The 95% C.I. on the prediction estimates ranged from 20.4% to 304.8%, underlining the difficulties of predicting the abundance of *Culicoides*.

**Conclusions:**

We found that significant spatial clusters of *Culicoides* moved around in a dynamic pattern varying between catch nights. This conforms with the modeling but was not explained by any of the tested covariates. The mean abundance within these clusters was up to 11 times higher for the Obsoletus group and 4 times higher for the Pulicaris group compared to the rest of the field.

## Background

Since the incursion of bluetongue virus into northern Europe and the subsequent discovery of Schmallenberg virus in the same region, *Culicoides* populations on farms have become important for epidemiological research. Species of the Obsoletus group and the Pulicaris group are suspected to play an important role in north European outbreaks of bluetongue and are found throughout northern Europe [1-5]. Recently, it was confirmed that species in the Obsoletus group can replicate Schmallenberg virus [6]. Many large-scale studies and transmission models have included spatial estimates of the abundance of *Culicoides* in Europe ([7-16]), but few studies have investigated the spatial pattern of *Culicoides* abundance on a local scale: In 1951, Kettle [17] found that the abundance of *C. impunctatus* decreased proportionally with distance to their breeding sites. This species is not dominant on farms but frequently associated with bogs (e.g. [18]). Later, Kettle [19] found indication of higher abundances of *C. impunctatus* and *C. pulicaris* L. near hosts (cattle, horses and humans). Garcia-Saenz *et al.*[20] found a positive correlation between the number of sheep near a light trap, and the number of female *C. obsoletus* caught in the trap. Rigot *et al.*[21] found that the abundance of different species of *Culicoides* were positively correlated with closeness to farms in Belgium. In a large scale study, Purse *et al.*[18] found that the abundance of adult *C. pulicaris* sensu stricto was correlated with vegetation indices, land use and elevation above sea level; *C. punctatus* abundance was correlated with the presence of sheep, temperature, land use and vegetation; and the abundance of *C. obsoletus* was only correlated with temperature. Also, the abundance of adult *C. impunctatus* was found to have a negative correlation with the presence of cattle, which might be because of their breeding sites (bogs) that are often located away from cattle. Remote sensing can be used to estimate the abundance of *Culicoides* (e.g. [8,18,22]), but provides only estimates of *Culicoides* abundance on a rough scale. In this study we take a novel approach, using local-scale abundance data to investigate possible spatial and temporal covariates for prediction of *Culicoides* abundance within a field.

Neighboring insect traps can be spatially dependent on each other (e.g. [23,24]), and Rigot et al. [25] found significant overlapping catching areas between 8 W Onderstepoort traps situated 50 meters apart. Thus it is necessary to take spatial autocorrelation into account. We developed conditional autoregressive (CAR) models for the abundance of two *Culicoides* vector species groups in order to account for the spatial dependency. For the first time, spatial autocorrelation is incorporated in a prediction model for *Culicoides* on a local scale, making trap catches spatially independent by including information from neighboring traps. Using this approach, a number of spatial covariates which have a significant impact in ordinary regression modeling, no longer appear significant and some temporal covariates become significant. At the same time we provide a method to deal with a lot of missing data in a spatial dataset by including second order neighbors when first order neighbors are missing. Furthermore, we estimate the spatial autocorrelation between trap catches and demonstrate the need to take it into account by incorporating it into statistical models. Lastly, we examine the abundance pattern not explained by the systematic part of the CAR models through cluster analysis.

## Methods

### Field data

**Figure 1:**
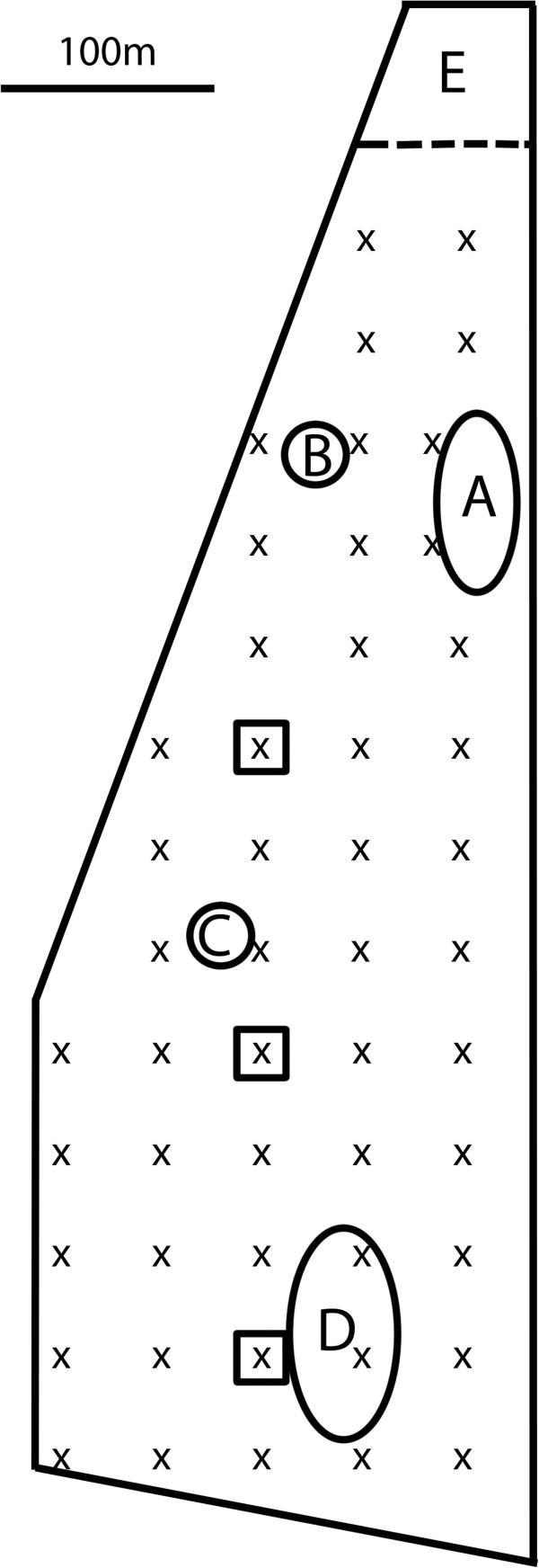
**Study site.** Outline of the study field with potential breeding sites (**A**, **B**, **C**, **D**) and the enclosure where the sheep were kept at night (**E**). Trap positions are marked with X and square boxes represent small enclosures for the transect experiment.

The study site was an approximately 750 m long and 250 m wide field grazed by sheep in Denmark (Figure 1, GPS coordinates: N55.3961, E12.1903), and the study period covered 7 weeks in June to August, 2009 (Table 1). The vegetation on the field was grasses and shrubs (about 10-30 cm height) and the field was completely surrounded by windbreaks consisting of trees and bushes (about 3-5 m height). No confounding light sources outside the field were visible at night. The surroundings were agricultural fields, except in the southern end and the north-western end of the field where there was tree cover. Fifty CDC Mini UV-light traps (John W. Hock, USA) were set up at a height of 180 cm in heavy metal gallows in 50 by 50 meter grid points covering the study field to measure the abundance of *Culicoides*. The grid size was chosen to sample the field evenly with little potential overlap of trap ranges [20]. For convenience we chose the CDC type traps and not the more commonly used Onderstepoort type trap. The CDC type traps are ideal for operation in the field using a 6 V battery and equipped with a photoswitch to save battery during the day when *Culicoides* are inactive. The traps turn on automatically at dusk and off at dawn. During the study period, 260 sheep (25-30 kg) had access to the whole field during the day, and were confined to a small enclosure in the northern end of the field before dusk until after dawn. This ensured that host animals were not present on the field at night and enabled a precise measure of the distance from each trap to the host animals.

**Table 1:** **The total number of *Culicoides * caught on each successful catch night and the temporal covariates**

**Date of sampling night**	**20.07.09**	**21.07.09**	**27.07.09**	**03.08.09**	**04.08.09**	**06.08.09**	**17.08.09**	**18.08.09**
Obsoletus group total	4	872	316	173	522	612	2	93
Obsoletus group min/max	0/1	2/79	0/68	0/106	1/79	2/48	0/1	0/20
Pulicaris group total	15	8015	1524	750	621	952	4	190
Pulicaris group min/max	0/5	18/914	5/323	7/128	0/65	6/80	0/2	0/27
Wind speed (m/s)	1.4	2.7	1.4	2.4	0.5	0.2	2.9	2.5
Turbulence	0	0	0	0.2	0	0.2	0	0
Temperature (Celcius)	14.8	13.6	14.9	18.1	15.4	15.3	16.6	13.7
Mean humidity (%RH)	84.4	88.3	77.5	85.0	94.6	84.5	90.5	77.3
Precipitation (mm)	0.2	0.2	0	0.6	3.6	0	0	0.2
**Date of sampling night**	**21.08.09**	**24.08.09**	**25.08.09**	**27.08.09**	**28.08.09**	**31.08.09**	**03.09.09**	**04.09.09**
Obsoletus group total	95	29	427	1086	1	253	2	1
Obsoletus group min/max	0/18	0/12	2/58	24/176	0/1	0/44	0/1	0/1
Pulicaris group total	223	33	817	1745	8	260	5	4
Pulicaris group min/max	0/23	0/9	3/139	44/166	0/4	0/37	0/2	0/2
Wind speed (m/s)	3.3	1.8	3.7	0.9	0.1	1.3	0.9	5.5
Turbulence	0.5	0	0	0	0	0	0.1	0
Temperature (Celcius)	19.9	15.9	17.3	15.6	18.4	12.1	14.8	14.3
Mean humidity (%RH)	90.6	78.7	81.9	77.5	82.0	80.2	87.6	87.0
Precipitation (mm)	0	0	0	0	0	0	0	0.2

On the underlined dates, half of the samples were not analyzed.

Four potential breeding sites (A-D on Figure 1) for the Pulicaris group were subjectively identified on the field [26]: Site A was a shallow assembly of water without any boundary vegetation and a 1-3 meter broad mud zone; Site B was an old marl pit with shallow water, heavily shaded by dense thicket with trees; Site C was a small pond with reed along the steep edges; site D was a muddy area on the field with small temporary water bodies. Throughout the study period, twenty to fifty (according to area size) mud samples (97 mm in diameter) were taken weekly from each potential breeding site and kept in emergence chambers at an indoor facility (following [27]) to confirm breeding. Outside the potential breeding sites, an additional 50 soil samples were taken weekly at randomly generated coordinates to screen for Obsoletus group breeding sites and for unexpected breeding sites of the Pulicaris group. We did not target breeding sites of the Obsoletus group as they are poorly investigated. They are associated with factors that are difficult to include in a model such as dung heaps and leaf litter, but this topic is still largely uncovered [28-30].

On three nights, sheep were placed in a transect in the middle of the field to test the attraction effect of a few sheep compared to the flock. In three transect points, two sheep were placed together in a 3 by 3 meter enclosure under a light trap (see Figure 1). The distance between transect points was 150 meters. During the study period, a weather station (Davis Vantage Pro 2) with a data logger was set up to record temperature, precipitation, humidity, wind speed and wind direction at 5 minute intervals. It was placed in the middle of the field to keep away from interfering vegetation. Light traps were emptied at dawn, and the caught *Culicoides* were preserved in 70% ethanol. The samples were analyzed under a dissection microscope, and sorted to species group and sex following Campbell and Pelham-Clinton [31]. Only females of the Obsoletus group (comprising *C. obsoletus*, *C. scoticus*, *C. chiopterus* and *C. dewulfi*) and the Pulicaris group (here comprising *C. pulicaris* and *C. punctatus*), were included in this analysis. We only considered the two dominant species in the latter group since other members of this group are rare in farm areas (pers. obs.) and not identified as a disease vector in this region. Due to time constraints, on 8 of the catch nights we only counted 50% of the trap catches. On these catch nights (the dates are underlined in Table 1), every second sample, chosen in a checkerboard pattern, was analyzed. All 16 nights were included in the models.

To deal with a high number of low catches we stabilized the observations by transforming the numbers with the natural logarithm prior to analysis, log(x+1). Thus for low numbers the observations will converge towards 1 instead of zero. For simplicity, we here denote the transformation as log(X) in the equations.

### Temporal covariates

Only weather records during the flight periods of *Culicoides* (assumed to be one hour before to three hours after sunset and two hours before to one hour after sunrise) were used in the analysis because we assume that the trap catches were only directly affected by the weather in this time interval. Mean temperature, humidity and wind speed measurements recorded on the field during the flight periods were included directly as covariates. Precipitation was summed over each flight period and included as a covariate. As an estimate of the wind turbulence, changes in wind direction was defined in steps as a minimum change of wind direction of 22.5 degrees. The highest number of steps that the wind direction changed in either 5 or 10 minute intervals, measured within each flight period, was calculated. As each catch night consisted of two flight periods, the mean of the two highest step change numbers for each flight period was used as the turbulence covariate for each catch night.

### Spatial covariates

The Euclidean distance from each trap to the sheep enclosure was used as a covariate (Table 2). The inverse distance, squared inverse distance, log distance and the square of the log distance were also included in the initial models. We hypothesized that *Culicoides* could take advantage of shelter from the wind behind windbreaks surrounding the field. To construct this effect of windbreaks, the angle difference between the wind direction and the windbreak angle was found. The covariate was then equal to sinus to the angle, resulting in full effect of windbreaks perpendicular to the wind direction, and no effect of windbreaks parallel to the wind direction. Furthermore, the effect of a windbreak was only included if the wind blew towards the field through the windbreak. The windbreak effects were then multiplied by the inverse distance from each trap to the respective windbreaks. For each trap, the sum of all windbreak effects was used in the analysis. An effect of sheep scent was modeled in a similar way, using the sine function on the angle difference between the wind direction and the fence separating the sheep from the field. This corresponded to the odor-seeking function used in the model of Sedda *et al.*[15]. On the three catch nights where sheep transects were set up, the inverse Euclidean distance from each trap to the transect points was included as a covariate. The inverse squared distance from each trap to the nearest breeding site was tested to account for the effect of breeding sites. The following interactions between covariates were also tested: distance to sheep and windbreak effect, sheep scent effect and windbreak effect, wind speed and windbreak effect, wind speed and sheep scent effect. Squared relationships were included to allow for non-linear effects. A systematic effect of each catch night was also included in the model.

**Table 2:** Mean, standard error and ranges of spatial covariates in the models

	**Mean**	**Variance**	**Range**
**Distance to**	390	30997	38 - 653
**Sheep (m)**			
**Breeding site effect**	4.7 · 10^−4^	9.6 · 10^−7^	3.6 · 10^−5^ - 5.1 ∗ 10^−3^
**Windbreak effect**	0.048	0.019	0.001 - 1.383
**Sheep scent effect**	4.5·10^−4^	6.97·10^−6^	0.000 - 0.041

### Ordinary regression modeling

We first build a linear regression model for each of the two species groups, using backwards 1-step reduction from a model including all covariates:

(1) $$\text{log}(X)∼\beta^{T}Z+\in$$

Where the log of the abundance of *Culicoides* (*X*) is determined by covariates *Z* and their coefficients *β* (where *β*^*T*^ signifies the matrix transpose of *β*), and a residual error term *∈*. Model reduction was performed with the likelihood ratio method, and covariates that did not contribute significantly (p ≥ 0.05) were excluded. After model reduction, all excluded covariates were tested again by forward selection, with the test sequence defined through the Akaike Information Criterion (AIC) [32]. These models treated the trap catches as stochastically independent of each other and hence ignored potential spatial autocorrelation (clustering). All regression modeling was carried out in R 2.14.2 (http://www.r-project.org).

### CAR modeling

To account for the spatial autocorrelation within the dataset, a conditional autoregressive model (CAR) model was constructed for each species group by assuming spatial dependence in the model (1) as described in the following. The model estimation and test procedure is described in Additional file 1.

In order to transform these spatially dependent observations into a series where standard estimation techniques could be applied, the traps were first listed in a specific sequence, the *conditioning series*, starting with the trap in the upper right corner of the field and continuing straight down (see Figure 1), then moving left along the bottom of the field, one step up to the next trap and continuing straight up, then left along the top of the field and so on. For these sequential data, the following model was defined:

(2) $$\text{log}(X)∼\beta^{T}Z+\varphi (\rho )N+\in$$

**Figure 2:**
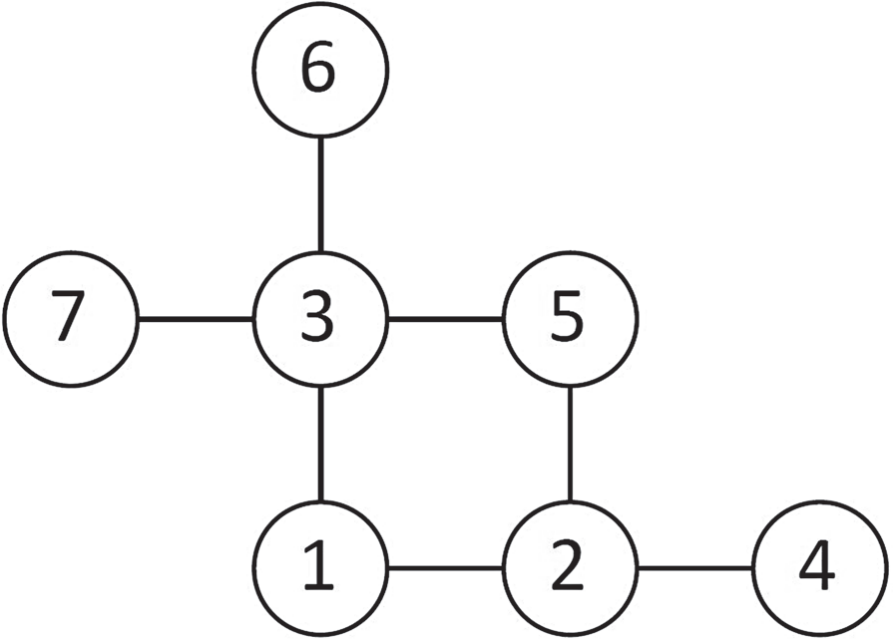
**Neighbors.** A scheme of the relationship between a trap (position 1) and its first order neighbors (positions 2 and 3) and second order neighbors (positions 4, 5, 6 and 7). This diagram covers all possible situations because the autocorrelation is estimated by successively conditioning, meaning that only neighbors for which the trap catch have not been conditioned before will be counted as neighbors.

Where the log of the abundance of *Culicoides* (*X*) is determined by the following components: The effect parameter matrix *β*, the vector of covariates *Z*, and the correlation matrix *φ*(*ρ*) capturing the effect of neighbors as a function of the spatial autocorrelation *ρ*, multiplied by the model’s residual values *N* for the specific neighbor configuration for each trap catch (for neighbors with higher index in the conditioning series). *β*denotes the residual error term. Equation (2) was based on the theoretical spatial autocorrelation framework using block design by Besag [33], and by definition assumes that each observation is independent of all other observations given the first order neighbors, when these are all present. First order neighbors to a trap (with the trap in position 1 on Figure 2) comprised all trap catches at 50 meters distance to the trap on the same catch night (position 2 and 3 on Figure 2). Second order neighbors were defined, for use in the estimation process when first order neighbors were missing, as first order neighbors to a first order neighbor, but not identical to the original trap (e.g. position 5, 6 and 7 are second order neighbors to position 1 through position 3 on Figure 2). The correlation between the traps and any first order neighbor was modeled as a constant *ρ*≥0. This, together with the requirement of conditional independence, defined the correlation structure between all traps, and thus *φ*(*ρ*) in equation 2, uniquely. For example, the correlation between a trap and a second order neighbor along a line transect was then *ρ*^2^. Thus the model implies exponentially decreasing dependence between traps along line transects if first order neighbors are missing. This model is different from a normal CAR model in that the regression is weighted with different variances for each spatial neighbor configuration. The configuration of first and second order neighbors to each trap, and thus *φ*(*ρ*), varies considerably in this analysis due to many missing observations. The standard error of *ρ* was estimated through the Fisher Information ([34]).

To evaluate the performance of the CAR models, the models were examined for significant spatial clusters in the residuals using a normal distribution model in SaTScan v. 91.1.1 (http://www.satscan.org). For each catch night and each species group, the model residuals were tested for circular or elliptic hotspots or coldspots, without penalty for elliptic clusters and allowing multiple hotspots. Each scan was run for 9999 iterations, testing for significant clusters at the 5% level. To investigate the spatial autocorrelation pattern not explained by the systematic part of the CAR models, we adjusted the observations for the significant effects found in the CAR models and then tested each catch night for significant clusters in SaTScan. Ordinary regression model and CAR model fit were tested by plotting the distribution of residuals and by quantile-quantile-plots.

No sheep were harmed in this study. Permission to move the sheep was obtained by the owners.

## Results

During the study period, successful catches from 16 nights, consisting of 530 trap catches, were included in the analysis. A total of 19,654 female *Culicoides* were counted: 15,166 from the Pulicaris group and 4,488 from the Obsoletus group (Table 1). The parameter ranges within the active period were: mean temperature: 12.1 – 19.9 degrees Celsius, mean wind speed: 0.08 – 5.47 m/s, precipitation: 0-3.6 mm, Relative humidity: 54-100%. The distance from each trap to the sheep was 38 – 653 meters and the distance to the nearest breeding sites 1-45 meters. Catches were excluded if the sheep broke through the enclosure during the night; the trap was damaged or not operating properly.

A total number of 208 *Culicoides* spp. hatched from the emergence chambers, of which 16 were from the Pulicaris group and none were from the Obsoletus group. The other species that hatched were mostly *C. pictipennis* and *C. festivipennis*. From breeding site A, 24 *Culicoides* spp. (none were from the Pulicaris group) emerged from 350 soil samples. From the shaded breeding site B (Figure 1) no *Culicoides* but many Psychodidae spp. emerged from 140 soil samples. From breeding site C, 152 *Culicoides* spp. (of which 13 were from the Pulicaris group) emerged from 140 samples. From breeding site D, 32 *Culicoides* spp. (of which 3 were from the Pulicaris group) emerged from 140 samples. No *Culicoides* emerged from the 350 random samples on the field, indicating that Pulicaris group breeding sites were confined to the identified breeding sites and that the Obsoletus group did not emerge on the field during the study period. Distance to breeding sites A, C and D was included in the modeling procedure as they were found to be breeding sites for *Culicoides*.

The temporal autocorrelation between sampling nights was tested in the CAR models, and in both models it was found to be insignificant (Obsoletus group model: p=0.51, Pulicaris group model: p=0.76). The spatial autocorrelation was highly significant (p-values: Obsoletus group model: p<0.0001, Pulicaris group model: p<0.0001), and was estimated to be 0.41 (+/-0.09) for the Obsoletus group model and 0.235 (+/-0.09) for the Pulicaris group model for traps placed with 50 m distance. The residual variance in the Obsoletus CAR model was 0.69 and in the Pulicaris CAR model 0.65 (Table 3).

**Table 3:** Significant coefficients from the models

	**Ordinary regression**				**CAR models**			
	**Obsoletus group**		**Pulicaris group**		**Obsoletus group**		**Pulicaris group**	
**Intercept**	0.73		0.43		-349		-386	
**Distance to sheep**	−4.5·10^−3^	***	−3.4·10^−3^	***	−4.7·10^−3^	***	−4.05·10^−3^	***
**Distance to sheep**^2^	6.0·10^−6^	***	5.7·10^−6^	***	6.3·10^−6^	***	6.42·10^−6^	***
**Precipitation**	NS		NS		−66.2	***	−73.40	***
**Turbulence**	NS		NS		−186.2	***	−206.6	***
**Humidity**	NS		NS		1.06	***	1.19	***
**Temperature**	NS		NS		39.95	***	43.91	***
**Temperature**^2^	NS		NS		−1.27	***	−1.40	***
**Wind speed**	−7.6·10^−4^	*	NS		1.84	***	2.27	***
**Wind speed**^2^	NS		NS		−0.18	**	−0.23·10^−2^	***
**Sheep transect**	NS		0.51	*	NS		0.4794	*
**Windbreaks**	−0.28	*	−9.7·10^−2^	***	NS		NS	
**Sheep scent**	-7.116	***	0.81	*	NS		NS	
Windbreaks * Sheep scent	4.8·10^3^	***	2077	*	NS		NS	
Wind speed * Sheep scent	-84.96	*	NS		NS		NS	
**Catch night 21.07**	2.68		4.84		-0.56		1.03	
**Catch night 27.07**	2.06		3.60		-4.24		-3.32	
**Catch night 03.08**	1.09		3.05		59.64		67.31	
**Catch night 04.08**	1.96		2.22		221.60		245.30	
**Catch night 06.08**	2.37		2.81		21.86		24.59	
**Catch night 17.08**	0.01		-0.04		-9.51		-10.76	
**Catch night 18.08**	0.73		1.09		-5.48		-5.92	
**Catch night 21.08**	1.34		2.11		108.80		120.60	
**Catch night 24.08**	0.36		0.38		NA		NA	
**Catch night 25.08**	2.72		3.17		NA		NA	
**Catch night 27.08**	4.11		4.56		NA		NA	
**Catch night 28.08**	-0.07		0.02		NA		NA	
**Catch night 31.08**	1.09		1.26		NA		NA	
**Catch night 03.09**	-0.05		-0.08		NA		NA	
**Catch night 04.09**	NA		-0.06		NA		NA	
**Residual variance**	0.68		0.65		0.69		0.65	

Insignificant covariates are denoted with “-”. *, p < 0.05; **, p < 0.01; ***, p < 0.001, NS, not significant; NA, no estimate. Catch nights were tested together, and the significance is shown at each first catch night. The p-values for the main effects includes the removal of both main effects and any interactions with this. No significances are given for the effect of catch nights, which were included as a systematic effect in all models.

The ordinary regression models without spatial autocorrelation identified more significant spatial covariates than the CAR models did, and the CAR models identified more temporal covariates than the ordinary regression models (Table 3).

**Figure 3:**
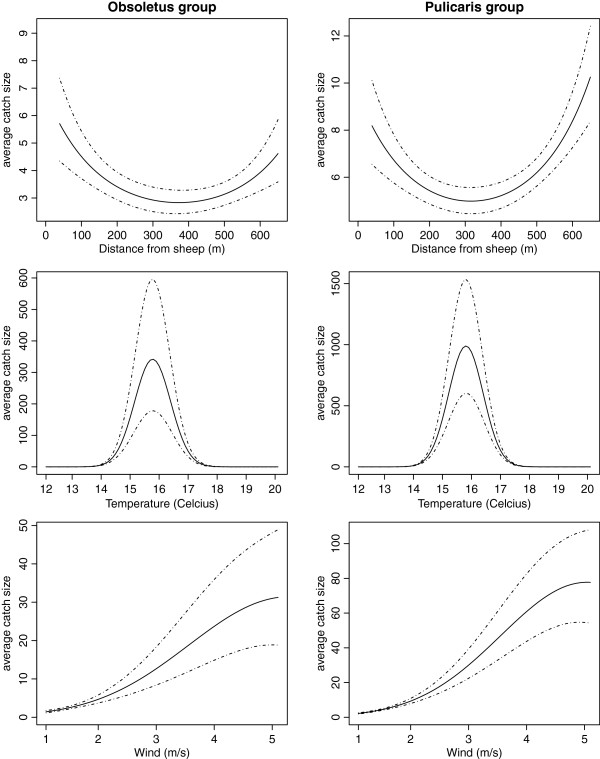
**CAR model effects.** General effect of the distance to the sheep, temperature and wind, resulting from the CAR models. Plots show the mean effects in the investigated intervals with 95% confidence intervals. The functions are shown in the investigated interval and the curves will be vertically shifted between catch nights. For both species groups, the level of abundance is above the 95% confidence limits for the distance with minimum catch up to 200 meters from the sheep. Also for both groups, there is a peak activity at just below 16 degrees.

The CAR models for both species groups showed increased abundance of *Culicoides* near the sheep (t-test p-values for both models: distance to sheep < 0.001, squared distance to sheep < 0.001). The mean abundance for the Obsoletus group was approximately twice as high near the sheep as 372 meters away where the minimum abundance level was found (Figure 3). The Pulicaris group abundance was approximately 1.5 times higher near the sheep than at 316 meters distance where the minimum abundance level was found. For both species groups, this effect was significant until 200 meters from the sheep, judged by visual inspection of the confidence limits on Figure 3. For both species there was an increase in the abundance estimate from 300 to 650 meters distance.

For both species groups we found a significant positive effect of humidity and a significant negative effect of turbulence and precipitation (Table 3). There was also a significant effect of temperature and wind speed including their squared terms. The temperature effect showed peak abundance at just below 16 degrees Celcius and the wind speed surprisingly showed a positive effect with increasing wind speed between 1 and 5 m/s (Figure 3).

Only the CAR model for the Pulicaris group identified a significant effect of the transect of sheep on three catch nights. The effect of the inverse Euclidean distance to the small enclosures with pairs of sheep is positive, meaning that the Pulicaris group abundance is higher close to the pairs of sheep.

We tested for clusters in the residuals of the CAR models to check if the spatial autocorrelation was fully extracted in the models. In the residuals of the Obsoletus group CAR model we found two clusters (p=0.0045 and 0.0006) on the nights of 28.08 and 31.08. We also found two clusters (p=0.0375 and 0.0427) in the Pulicaris group CAR model on the nights of 06.08 and 31.08.

**Figure 4:**
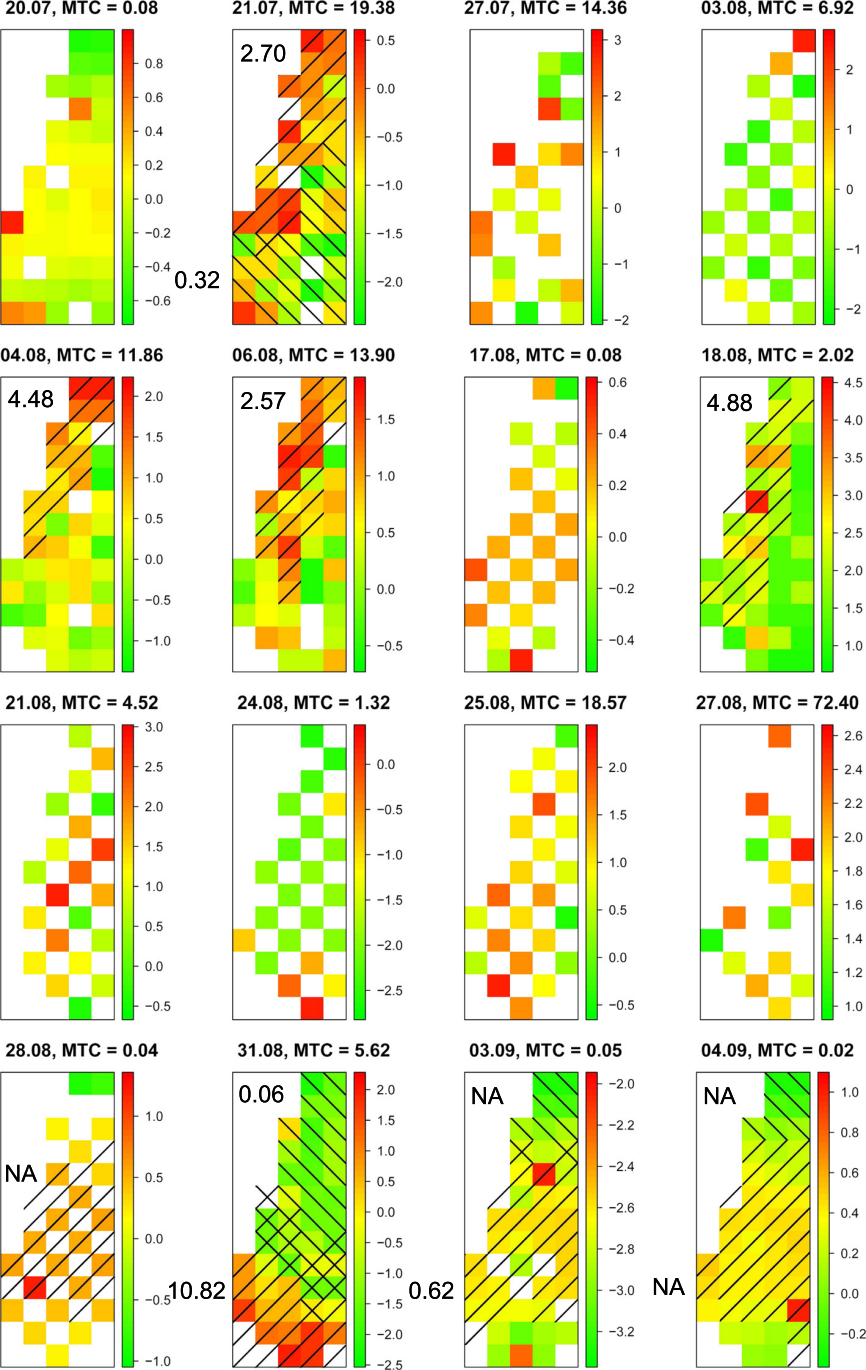
**Obsoletus group abundance pattern.** Visualization of the spatial clustering left in the data when the CAR model effect has been subtracted. Maps show the log of trap catch size for the Obsoletus group each catch night without the effect of distance to host animals. Traps that are included in significant hotspots are right-hatched and those in significant coldspots are left-hatched. The mean abundance ratio is noted for each cluster. MTC = mean trap catch per catch night. Note that the hotspots are moving around from catch night to catch night, and that some of the hotspots are similar to Figure 5. The low hotspot ratio on the night of the 3rd September is an artifact caused by low catch numbers.

**Figure 5:**
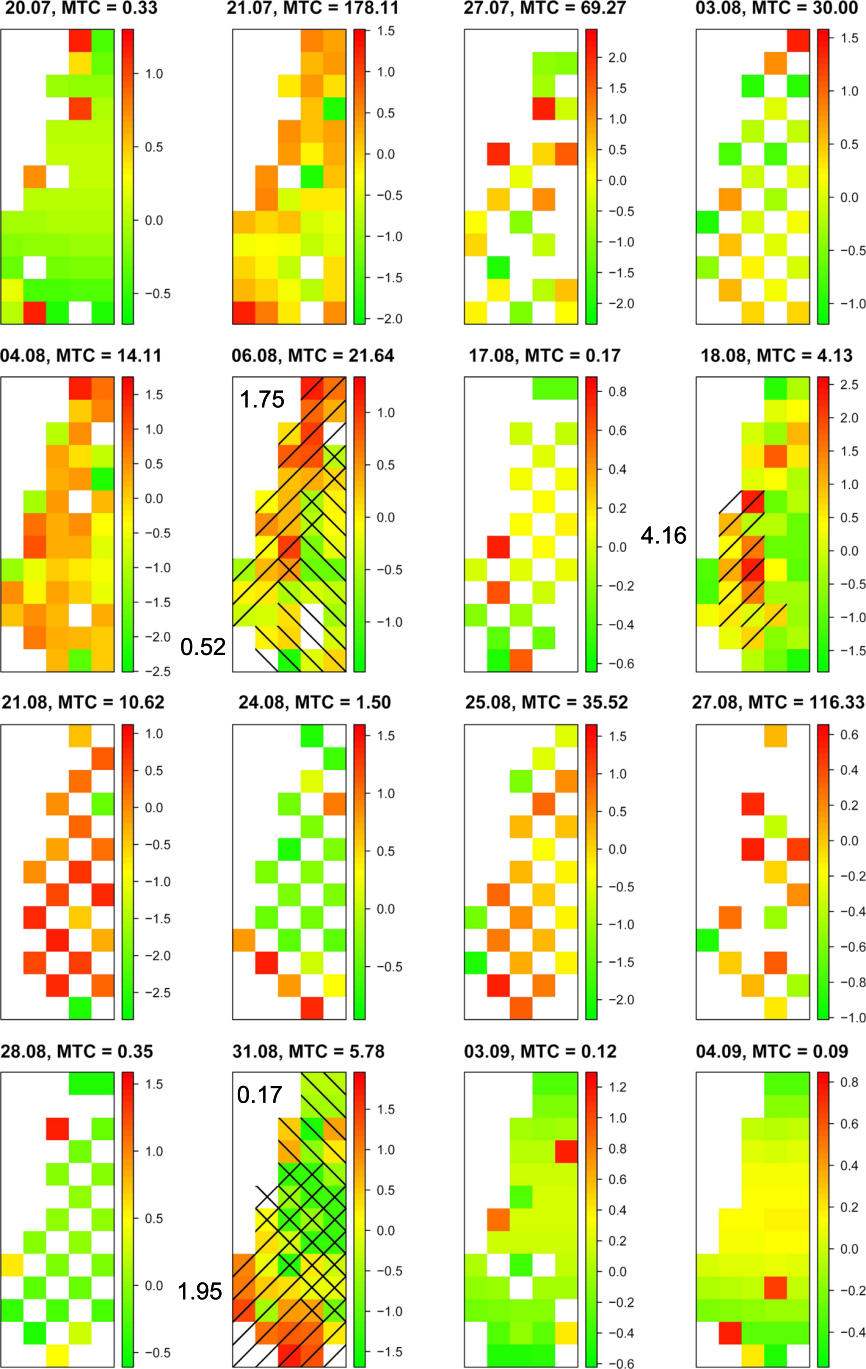
**Pulicaris group abundance pattern.** Visualization of the spatial clustering left in the data when the CAR model effect has been subtracted. Maps show the log of trap catch size for the Pulicaris group each catch night without the effect of distance to host animals. Significant traps that are included in significant hotspots are right-hatched and those in significant coldspots are left-hatched. The mean abundance ratio is noted for each cluster. MTC = mean trap catch per catch night. Note that the hotspots are moving around from catch night to catch night, and that some of the hotspots are similar to Figure 4.

To investigate the spatial clustering pattern of vector abundance not explained by the systematic covariates in the CAR models, we subtracted the CAR model effects from the observations and tested for clusters using SaTScan. This procedure extracted the significant effects found in the CAR models without extracting the spatial clustering from the data, allowing us to examine the unexplained abundance pattern. Eight significant hotspots (mean trap catch ratios for catches within versus catches outside clusters: 2.70; 4.48; 2.57; NA; 10.82; 0.62; NA, where NA indicate an error caused by zero catches) and four significant coldspots (ratios: 0.32; 0.06; NA; NA) were found in the Obsoletus group data. In the Pulicaris group data, three hotspots (ratios: 1.75; 4.16; 1.95) and two coldspots (ratios: 0.52; 0.17) were identified (Figures 4 and 5). In the Obsoletus group, four of the hotspots were found in the northern part of the field, three in the middle and one in the southern part. Also for this group there were three coldspots in the northern part and one in the southern part. One of the hotspots in the Pulicaris group data was found in the northern part, one in the middle and one in the southern part of the field. The two significant coldspots were located both in the northern and the southern part. Some of the traps were included in both hotspots and coldspots, which is a consequence of the SaTScan method forcing the cluster to be circular or elliptic. This highlights the short distance between hotspots and coldspots on the field. The significant hotspots and coldspots are placed similarly but not identically in the two species groups.

## Discussion

We tested the observations for clustering without the effect of host animals to investigate the spatial clustering pattern not explained by the systematic covariates in the CAR models. It revealed a dynamic pattern with higher *Culicoides* abundance in different places, varying between catch nights, so clusters were moving around on the field (Figures 4 and 5). This is consistent with the CAR modeling, and implies that one or more yet unidentified factors influenced the *Culicoides* abundance in a spatial pattern that changes each night. The ratios of the significant hotspots show that the mean abundance of the Obsoletus group in a significant hotspot was 0.62-10.82 times higher than the rest of the field (Figure 4), and 1.75-4.16 times higher for the Pulicaris group (Figure 5). This result is striking and can seriously impact field studies of *Culicoides* abundance. Since no known factor could explain this dynamic pattern, it will cause noise in abundance studies. The best way to to take account for this is to conduct large-scale studies with many traps and locations reducing the noise from the spatial clustering. It is not possible to obtain a reliable measure of the level of abundance in an area by using a single trap. However, this does not mean that national or regional scale predictive abundance models are invalid if they are based on just one trap per farm. If a large number of farms are sampled, the general relationship between environmental factors and the mean abundance can still be quantified. Such models may therefore be able to predict a mean trap collection on farms associated with a specific combination of environmental covariates (e.g. [8,11,12]). But if the same models are used to predict catch sizes in a trap at a specific farm it may result in very large residuals as a result of the large spatial variation in abundance on the same farm.

In this study we found that the spatial autocorrelation between traps was highly significant. This means that if a trap catches more than expected, another trap close by is also likely to catch more than expected. For the Obsoletus group the spatial autocorrelation was 0.41. We explored this further (using equation (5), see Additional file 1), to have a look at the relation between two traps, A and B, with 50 m distance. For an expected level of abundance at 100% in both traps, if trap A catches 20% more than expected, then trap B will be expected to catch 7.8% more. If trap A catches 50% more than expected, trap B will be expected to catch 18.1% more. For the Pulicaris group the spatial autocorrelation was 0.235. Using the same scenarios, trap B would catch 4.4% and 10.0% more than expected, respectively. The spatial autocorrelation means that traps placed close to each other do not provide independent estimates of abundance. The true variance in abundance will therefore be underestimated unless traps are widely separated. This has to be taken into account when using more than a single trap at a site.

The CAR models should extract the spatial clustering from the data and therefore leave no significant clusters in the residuals. However, we found two clusters (p=0.0045 and 0.0006) in the residuals of the Obsoletus group CAR model. The first cluster is on the night of the 28.08.2009 where only one specimen from the Obsoletus group was caught on the entire field, and thus we ascribe this cluster as an artefact. The second cluster in the Obsoletus group CAR model residuals on the night of the 31.08.2009 is also highly significant. We performed the parameter estimation again without this catch night and found similar estimates of the effects (data not shown). Thus we conclude that this model violation does not influence the general validity of the model. Two clusters were found in the Pulicaris group CAR model residuals with p-values only just below the significance level (p=0.0375 and 0.0427). Therefore we do not doubt the general applicability of this model either. Furthermore, the SaTScan analysis used to detect clusters is not able to deal with the varying variance included in the model residuals created by differing neighbor configurations, making this test very rigid.

From the two CAR models, the residual variance was estimated to be 0.69 and 0.65 (Table 3). We can use this variance to estimate the general 95% confidence intervals of the abundance estimates. Thus the 95% interval for Obsoletus group CAR model ranged from 20.4% to 304.8% of the predicted catch size. For the Pulicaris group CAR model the interval ranged from 22.6% to 289.4%. This highlights the huge variation in the catches. Estimates of vector abundance based on single traps are expected to vary dramatically depending on the exact position chosen for the trap. This high uncertainty associated with abundance estimates based on single traps needs to be taken into account when modeling the abundance of *Culicoides* on a greater scale and in simulation models of vectorborne disease that rely on vector abundance estimates.

The estimates of the significant effects in the models are fairly similar between the two species groups (Figure 3, Table 3). This supports the results of the models and indicates that the effects found may be general for species of *Culicoides*. Especially the significant temporal covariates, which may be general for *Culicoides* because they are not influenced by host preferences.

The dynamic pattern is also fairly similar between the two species groups. Surprisingly, three of the significant hotspots for the Obsoletus group and two for the Pulicaris group were found in the southern part of the field, away from the sheep. A possible explanation for this is swarming behavior. Downes observed in 1955 [35] that different species of *Culicoides* swarm above certain markers such as cow dung, a dark cloth or other conspicuous objects. Both the Obsoletus group and the Pulicaris group have been observed swarming, and it is likely that swarming can blur the general abundance pattern. Very few males were caught in the light traps in this study, and they seemed to be correlated with high female abundance (data not shown), which could also indicate swarming behaviour.

Similar to the results from other studies [20,21,36], we found a significant effect of the vicinity of host animals for both the Obsoletus group and the Pulicaris group. In a study of Calvete *et al.*[11], traps were placed within 30 m from each farm to obtain estimates of the abundance of *Culicoides*, and Goffredo and Meiswinkel [37] pointed out that when monitoring *Culicoides*, light traps should be placed in the near vicinity of vertebrate hosts. This is supported by the present study where we quantified the effect of host animals. We found that traps placed near host animals increased the overall vector abundance with approximately 50% - 100% compared with 300-400 m away from the host animals. However, we also found an increased level of abundance for both species groups in the southern part of the field. This could be an artefact in the simple two-parameter model construction, or it could indicate a depletion of *Culicoides* abundance around the host animals. In the latter case, the abundance level is normal again at 650 m distance from the host animals. An alternative explanation could be that this effect is caused by the small forest area in the southern part of the field.

This pattern is relevant for other studies of the abundance of *Culicoides*. Traditionally, *Culicoides* monitoring programmes are carried out running a single trap on each farm near host animals. Calvete *et al.*[11] mentions that traps were placed within 30 m from the hosts to ensure a high catch. Goffredo and Meiswinkel [37] suggest that traps are placed in the vicinity of hosts for monitoring programmes. We suggest, that the trap placement should be standardized or adjusted with regards to the distance to host animals because the distance to the hosts impacts directly on the trap catch. For instance, if placement of the traps just next to the host animals is impossible, all traps in a study should be placed at the same distance to obtain comparable measures at different farms. Alternatively, if one trap is placed sub-optimally at for instance 300 meters distance from the host animals, catches of the Pulicaris group made here should be adjusted up by 150%.

We also found a significant effect of the sheep placed in transects on the field for the Pulicaris group. This emphasizes that this species group is more abundant where the host animals are, and that even two sheep can have an impact on the abundance of this species group as found by Garcia-Saenz *et al.*[20]. It also underlines the fact that *Culicoides* can find any small group of host animals regardless of other groups of hosts nearby, which makes them very efficient disease vectors.

The temperature was significant for both species groups with peak abundance at 16 degrees Celcius and no effect below 14 degrees or above 18 degrees (Figure 3). This is in concordance with Conte *et al.*[12] who found that the minimum temperature for activity of the Obsoletus Complex was 14.2 (13.9–14.6) degrees Celcius. Garcia-Saenz *et al.*[20] found no significant effect of temperature on the abundance of *Culicoides*, but Carpenter *et al.*[38] found a peak biting rate at 21 degrees. The latter study included catches at temperature up to 29 degrees, which was not possible to include in the present study.

The humidity was found to have a positive significant effect on the abundance of both species groups. This is in concordance with Carpenter *et al.*[38] who found a positive correlation between humidity and *Culicoides* abundance. Carpenter *et al.*[38] and Baylis *et al.*[39] also found a positive effect of humidity on the abundance of the Obsoletus group. Turbulence had a significant negative effect in the CAR models for both species groups. Carpenter *et al.*[38] also found this significant effect. In the present study we also found that precipitation had a negative effect on the *Culicoides* abundance. This contrasts with the findings of Blackwell [40] who found a positive effect of rain on catches of *C. impunctatus*.

We found a significant effect of wind speed and its quadratic term for both species groups. When plotting with confidence intervals, the abundance increases with the wind speed in the investigated interval (Figure 3). In contrast, Carpenter *et al.*[38] found decreasing abundance for wind speeds exceeding 3 m/s. A possible explanation of the findings in our study is that if the wind is weak and the *Culicoides* therefore have difficulties in determining the direction of hosts by scent, they are reluctant to waste energy on flying. Thus, within the investigated range of windspeed, higher wind speeds yield a higher abundance of active *Culicoides*.

No *Culicoides* emerged from breeding site B (Figure 1). This could be due to the thicket and trees shading the pond, which prevents the sun from heating up the mud to the necessary temperature for *Culicoides* to breed. The other three sunlit breeding sites were expected as breeding sites for *Culicoides* spp. In this study we used light traps to measure *Culicoides* abundance. Therefore the results may be influenced by bias of the trapping method such as variation in attraction for different species and for different lifestages of *Culicoides*[38,41-43]. Future trapping studies should ideally distinguish specimens to species level in order to determine the differences in the behaviour between species with regards to light traps.

The spatial autocorrelation, *ρ*, was found significant, meaning that it is necessary to take spatial clustering into account on this scale. Even on a larger scale, spatial clustering is important to incorporate in the modeling framework as shown by [16]. The temporal autocorrelation, *θ*, was found non-significant. This was expected since the intervals between catch nights ranged from 0 to 10 nights. The ordinary regression models identified more significant spatial covariates than the CAR models, effects which the CAR models discarded through the inclusion of local dependence given by the spatial correlation (Table 3). A possible explanation for the extra significant spatial covariates included in the ordinary regression models is that they compensate for the spatial clustering by including more explanatory covariates, and it should be noted that given the validity of the CAR model, these significances are type 1 errors, ie. false significances. This interpretation is further supported by the fact that the significant covariates shared by the CAR models and the ordinary regression models are fairly alike (Table 3). In the present study, the systematic effect of each catch night may have overtaken the effect of some of the covariates when few catch nights are sampled because non-spatial covariates will covary with catch night, which is a drawback of this type of model. However, the advantage is that we obtain more precise estimates of significant covariates corrected for the effect of spatial autocorrelation.

We used Besag’s block design to build the CAR models in this study [33]. Formulating the spatial autocorrelation as an exponentially decreasing correlation between neighboring traps we were able to include data points where all first order neighbors were missing by taking second order neighbors into account. This approach is useful in studies of grid measurements where many missing data are present.

The spatial autocorrelation between trap catches, *ρ*, accounts for other potential unknown covariates which were not spatially consistent between catch nights. However, if an unknown, spatially fixed factor influenced the abundance of *Culicoides*, the temporal autocorrelation, *θ*, would tend to be significant, indicating that some traps consistently caught higher numbers of *Culicoides*. But since the temporal autocorrelation was found insignificant and the spatial autocorrelation was found significant, there is no evidence for the presence of unknown spatially fixed covariates.

## Conclusions

We revealed a spatially varying pattern of abundance that varies between catch nights, where unpredictable hotspots caused the mean trap catch to be up to 11 times higher for the Obsoletus group and 4 times higher for the Pulicaris group. From the residual variance of the models we calculated that the 95% C.I. on the prediction of abundance is approximately 20% to 300%, which is important to consider when conducting large-scale studies. We found no significant spatial covariates determining the abundance of the studied species groups other than the distance to host animals and for the Pulicaris group this also included pairs of sheep placed in small enclosures on the field. Thus no low risk areas for placing host animals susceptible to bluetongue or Schmallenberg virus were identified on this scale because the abundance of *Culicoides* was indeed determined by the presence of host animals. We have demonstrated the importance of placing traps near the hosts when monitoring *Culicoides*, as we see a significantly increased abundance of *Culicoides* (up to 100%) in a radius of approximately 200 meters from the hosts. We also found significant positive effects of humidity and wind speed, significant negative effects of precipitation and turbulence. The optimum temperature for abundance of both species groups was found to be just below 16 degrees Celcius.

## Competing interests

The authors declare that they have no competing interests.

## Authors’ contributions

This project is a main part of the PhD project by Carsten Kirkeby at the Veterinary Institute at the Technical University of Denmark. Carsten Kirkeby conceived the study, carried out the planning, the field work, the analysis, contributed to the parameter estimation technique and wrote the manuscript. René Bødker participated in the planning, analysis and discussion of the results. Anders Stockmarr participated in the planning of the field work, provided the technique for the parameter estimation and took part in the analysis and the discussion of the results. Peter Lind participated in the discussion of the results. All authors read and approved the final version of the manuscript.

## Supplementary Material

Additional file 1 Description of CAR model estimation and testing procedures and the impact of spatial autocorrelation. Click here for file
