# Supplementary material for: Spatial abundance and clustering of Culicoides (Diptera: Ceratopogonidae) on a local scale
Source: Parasit Vectors. 2013 Feb 22;6:43. doi: 10.1186/1756-3305-6-43 (PMC3608001; doi:10.1186/1756-3305-6-43)
Supplement: Additional file 1 — Description of CAR model estimation and testing procedures and the impact of spatial autocorrelation. [file 1756-3305-6-43-S1.pdf]

S1 (Supporting Information 1)

CAR model estimation and testing

In order to estimate the parameters in the CAR model, we first set up a linear regression model corresponding to equation (1) including all covariates, with spatial dependence modeled through first and second order neighbors as described above. A product likelihood was found through successive conditioning of the full likelihood in the conditioning series, so that each trap catch was conditioned with all trap catches with higher index in the conditioning series. In the situation with missing first order neighbors, conditioning was performed on both first and relevant second order neighbors. The conditioning series impacted through first and second order neighbors with higher index, following the pattern in fig. 2 so that, using Besag's (1974) block design, only 1st order neighbors impact if none of these are missing, and 2nd order neighbors enters if any 1st order neighbors are missing. For example, if the 1st order neighbor in position 3 in Fig. 2 is missing, the 2nd order neighbors in positions 5, 6 and 7 will enter the list of neighbors on which conditioning is performed. If the 1st order neighbors at position 2 and 3 are both present, $\varphi(\rho)$ is a 1 x 2 matrix with values equal to $2\rho/(1+\sqrt{1+8\rho^2})$ at both entries. This transforms the model (1), with spatial autocorrelation, into the linear regression model for independent variables (2) by essentially including the spatial autocorrelation as a regression parameter. Parameter estimation was performed in a two-step procedure, where estimates of β were calculated conditionally on ρ, subsequently maximizing the profile likelihood for rho. First we fixed a value $\rho_0 >= 0$ for ρ. Then the effects of neighbor observations $\varphi(\rho_0)N$ in equation (2) was written as $\varphi(\rho_0)(\log(X_N) - Z_N^T\beta)$, were X_N is a vector containing the neighbor abundances, and Z_N is a matrix with each column containing the covariates for the corresponding neighbor. Now observe that:

$$\varphi(\rho)N = \varphi(\rho)(\log(X_N) - Z_N^T\ \beta) = \varphi(\rho)\log(X_N) - \beta^T Z_N\ \varphi(\rho)^T \tag{3}$$

To write the model (2) on a standard form with independent variables, (2) is re-written by splitting the term $\varphi(\rho)N$ as described in (3), subtracting $\varphi(\rho_0)\log(X_N)$ from (the log of) each observation and similarly subtracting $Z_N\varphi(\rho_0)^T$ from each set of covariates, thus transforming the model (2) back to a model of the same form as (1), but differing from an ordinary regression model in that variances for the observations are not identical, but differ as the neighbor configurations differ. Estimation of β for $\rho = \rho_0$ was then carried out by weighted linear regression with the variances acting as weights, thus taking within-neighbor correlation into account. In the event where successive conditioning yielded only approximate independence, because neither 1st nor 2nd order neighbors were observed in full in the configuration in fig. 2, full independence was

assumed for the estimation purpose. Taking the value of the likelihood function maximized for β this way as the value of the profile likelihood for rho at ρ_0, the estimation procedure was completed by maximizing the profile likelihood to obtain simultaneous estimates for β and rho that yielded the maximum value of the likelihood function.

To test if there was significant temporal autocorrelation between the catch nights in the dataset, we expanded the developed model to include the trap catch from the previous catch night as a regression parameter, W in (3), with the coefficient θ:

$$\log(X) \sim \beta^T Z + \theta W + \varphi(\rho) N + \epsilon \tag{4}$$

Estimation was then performed as described above. The CAR models were reduced sequentially with the likelihood ratio test at a 5% significance level, and a forward selection procedure similar to the ordinary regression analysis was subsequently performed. To finally test if the spatial autocorrelation was significant, we set ρ to zero and tested if this model performed significantly worse than the developed CAR model (significance level = 5%). The same procedure was used to test if the temporal autocorrelation was significant, setting θ to zero.

Impact of spatial autocorrelation

To investigate the impact of spatial autocorrelation on two traps, A and B, placed with 50 m distance, we use this formula to find the adjusted expected level of abundance in trap B:

$$A_{B|A} = \ E_B * (E_A/O_A)^{\rho} \tag{5}$$

Where A_B is the adjusted expected catch size of trap B given trap A, E_B is the general expected catch level of trap B, E_A is the expected catch level of trap A and O_A is the observed catch in trap A. ρ is the spatial autocorrelation at 50 m distance.

References

1. Besag J: **Spatial interaction and the statistical analysis of lattice systems**. *Journal of the Royal Statistical Society. Series B (Methodological)* 1974, :192–236.
2. Fisher R: **On the mathematical foundations of theoretical statistics**. *Philosophical Transactions of the Royal Society of London. Series A, Containing Papers of a Mathematical or Physical Character* 1922, **222**(594-604):309–368.
